# The importance of translational neuroscience in troubled times

**DOI:** 10.1093/braincomms/fcaf226

**Published:** 2025-07-03

**Authors:** Tara L Spires-Jones

**Affiliations:** Edinburgh, UK

## Abstract

Our editor discusses the importance of translational neuroscience, continued funding and international collaboration.

Welcome to Volume 7, Issue 4 of Brain Communications. As Editor of *Brain Communications*, a scientific journal that funds a charity promoting research, teaching and education in neurology and neuroscience, I feel compelled to speak out in support of science. In the last year, we have witnessed an unprecedented attack on science and scientists. These attacks are concerning, as they will stall or prevent scientific advances and damage the economy; we seem to be throwing away the seeds of the future crops of new knowledge. In the US alone, the scale of funding cuts is in the order of billions of dollars.^1^ Those funding cuts, along with cuts in training programmes and federally imposed prohibitions on international science, risk severe long-term harm to future advances. Immediately, it threatens to halt the amazing progress towards therapies in areas across neuroscience, from common diseases of old age, like Alzheimer’s disease, to rare paediatric fatal disorders, like Zellweger syndrome.

Our field of translational neuroscience is in a critical period of development. Over the past 150 years, advances in microscopy, molecular biology, genetics, and more recently, neuroscience, have led to an understanding of the fundamental properties of the nervous system. While we do not fully understand how neuronal networks produce amazing emergent properties—including learning, memory, complex behaviours and sense of self—there have been leaps in the understanding of cellular function that have started to translate into treatments for disorders affecting the brain. Analysis from the Global Burden of Disease Study 2021 indicates that neurological conditions are the leading cause of ill health and disability globally, with over 1 in 3 people affected worldwide.^2^ The only way we can reduce this burden is through research. None of the top 10 neurological conditions contributing to loss of health currently have medical cures, although there has been progress in treatments. For example, the first drugs that can slow the progress of Alzheimer’s disease have been approved in the past few years. However, these treatments only moderately slow cognitive decline, do not work for everyone, and come with significant costs and risks of side effects, resulting in a ‘regulatory rollercoaster’ with approvals in some parts of the world but not others.^3^ One of the key knowledge gaps that scientists in the Alzheimer’s field have been trying to address to develop better treatments is how gender, race and ethnicity influence disease pathogenesis in order to develop safer and more effective treatments that will work for more people affected by the disease (see, for example, two recent papers published in *Brain Communication*s^4,5^). This is not the time to delay work that promises the development of life-changing treatments.

In addition to advancing knowledge and medical progress, investment in scientific research boosts the economy. In the UK, the Government Department for Science, Innovation and Technology (DSIT) commissioned a report on the returns on public investment in research which estimates returns of around 40%, 6 years after investments are made, meaning for every £100 million the government invests, there is an increase of £40 million on annual gross value added in the private sector.^6^ This increase persists over time, boosting the economy. In the US, a study by the United for Medical Research estimated that in 2024 every dollar invested in the National Institutes of Health (NIH) resulted in a return of $2.56 in economic activity and that NIH funding produced over $90 billion in new economic activity.^7^

Science is a global game, and damaging science anywhere will hinder progress everywhere. There are data on paper citations demonstrating that papers with authors from more than one country have higher citation rates than single-country papers.^8^ Here, at *Brain Communications*, 6 out of our top 10 most highly cited papers have authors from more than one country, and 47% of our papers are international collaborations with authors from at least two different countries. We have published papers from authors in over 75 countries and similarly have reviewers contributing peer reviews from a wide geographical spread with over 59 countries represented.

How can one relatively small journal and editorial team help beyond continuing our core mission to publish robust translational neuroscience studies? We can at least add our voices and our data to reinforce and amplify the importance of what we do, emphasize the synergies and importance of international science, and articulate the corollary—that to impair science will preclude the advances that are necessary to help patients with devastating neurological diseases, both short term and long term.

The cover image for this issue comes from Crowley *et al*.^9^ and shows a uniform manifold approximation and projection (UMAP) which highlights the diversity of CD4^+^ T cells in the cerebrospinal fluid of people with multiple sclerosis.
